# Evaluation of linearly solvable Markov decision process with dynamic model learning in a mobile robot navigation task

**DOI:** 10.3389/fnbot.2013.00007

**Published:** 2013-04-05

**Authors:** Ken Kinjo, Eiji Uchibe, Kenji Doya

**Affiliations:** ^1^Neural Computation Laboratory, Graduate School of Information Science, Nara Institute of Science and TechnologyIkoma, Nara, Japan; ^2^Neural Computation Unit, Okinawa Institute of Science and TechnologyOnna-son, Okinawa, Japan

**Keywords:** optimal control, linearly solvable Markov decision process, model-based reinforcement learning, model learning, robot navigation

## Abstract

Linearly solvable Markov Decision Process (LMDP) is a class of optimal control problem in which the Bellman's equation can be converted into a linear equation by an exponential transformation of the state value function (Todorov, 2009b). In an LMDP, the optimal value function and the corresponding control policy are obtained by solving an eigenvalue problem in a discrete state space or an eigenfunction problem in a continuous state using the knowledge of the system dynamics and the action, state, and terminal cost functions. In this study, we evaluate the effectiveness of the LMDP framework in real robot control, in which the dynamics of the body and the environment have to be learned from experience. We first perform a simulation study of a pole swing-up task to evaluate the effect of the accuracy of the learned dynamics model on the derived the action policy. The result shows that a crude linear approximation of the non-linear dynamics can still allow solution of the task, despite with a higher total cost. We then perform real robot experiments of a battery-catching task using our Spring Dog mobile robot platform. The state is given by the position and the size of a battery in its camera view and two neck joint angles. The action is the velocities of two wheels, while the neck joints were controlled by a visual servo controller. We test linear and bilinear dynamic models in tasks with quadratic and Guassian state cost functions. In the quadratic cost task, the LMDP controller derived from a learned linear dynamics model performed equivalently with the optimal linear quadratic regulator (LQR). In the non-quadratic task, the LMDP controller with a linear dynamics model showed the best performance. The results demonstrate the usefulness of the LMDP framework in real robot control even when simple linear models are used for dynamics learning.

## 1. Introduction

When we want to design an autonomous robot that can act optimally in its environment, the robot should solve non-linear optimization problems in continuous state and action spaces. If a precise model of the environment is available, then both optimal control (Todorov, 2006) and model-based reinforcement learning (Barto and Sutton, 1998) give a computational framework to find an optimal control policy which minimizes cumulative costs (or maximizes cumulative rewards). In recent years, reinforcement learning algorithms have been applied to a wide range of neuroscience data (Niv, 2009) and model-based approaches have been receiving attention among researchers who are interested in decision making (Daw et al., 2011; Doll et al., 2012).

However, a drawback is the difficulty to find an optimal policy for continuous states and actions. Specifically, the non-linear Hamilton-Jacobi-Bellman (HJB) equation must be solved in order to derive an optimal policy. Dynamic programming solves the Bellman equation, which is a discrete-time version of the HJB equation, for discrete states and actions problems. Linear Quadratic Regulator (LQR) is one of the well-known optimal control methods for a linear dynamical system with a quadratic cost function. It can handle continuous states and actions, but it is not applicable to non-linear systems.

Recently, a new framework of linearly solvable Markov decision process (LMDP) has been proposed, in which a non-linear Bellman's equation for discrete and continuous systems is converted into a linear equation under certain assumptions on the action cost and the effect action on the state dynamics (Doya, 2009; Todorov, 2009b). In fact, the basis idea of linearization of the HJB equation using logarithmic transformation has been shown in the book written by Flemming and Soner and its connection to risk sensitive control has been discussed in the field of control theory (Fleming and Soner, 2006). Their study has been receiving attention recently in the field of robotics and machine learning fields (Theodorou and Todorov, 2012) because there exist a number of interesting properties in the linearized Bellman equation (Todorov, 2009b). There are two major approaches in LMDP: the path integral approach (Kappen, 2005a,b) and the desirability function approach (Todorov, 2009b). They are closely related and new theoretical findings are reported (Theodorou and Todorov, 2012), but there are some differences in practice. In the path integral approach, the linearized Bellman is computed along paths starting from given initial states using sampling methods. The path integral approach has been successfully applied to learning of stochastic policies for robots with large degrees of freedom (Theodorou et al., 2010; Sugimoto and Morimoto, 2011; Stulp and Sigaud, 2012). The path integral approach is best suited for optimization around stereotyped motion trajectories. However, an additional learning is needed when a new initial state or a new goal state is given. In the value-based approach, an exponentially transformed state value function is defined as the *desirability function* and it is derived from the linearized Bellman's equation by solving an eigenvalue problem (Todorov, 2007) or an eigenfunction problem (Todorov, 2009c; Zhong and Todorov, 2011). One of the benefits of the desirability function approach is its compositionality. Linearity of the Bellman equation enables deriving an optimal policy for a composite task from previously learned optimal policies for basic tasks by linear weighting by the desirability functions (da Silva et al., 2009; Todorov, 2009a). However, the desirability function approach has so far been tested only in simulation. In this study, we test the applicability of the desirability function approach to real robot control.

In order to apply the LMDP framework to real robot applications, the environmental dynamics should be estimated through the interaction with the environment. This paper proposes a method which integrates model learning with the LMDP framework and investigates how the accuracy of the learned model affects that of the desirability function, the corresponding policy, and the task performance. Although Burdelis and Ikeda proposed a similar approach for the system with discrete states and actions (Burdelis and Ikeda, 2011), it is not applicable to a continuous domain. We test the proposed method in two tasks. The first task is a well-known benchmark, the pole swing-up problem. We use linear and non-linear models for approximation of the environmental dynamics and investigate how the accuracy of the dynamics model affects the estimated desirability function and the corresponding policy. The second task is a visually guided navigation problem using our Spring Dog robot which has six degrees of freedom. The landmark with the LED is located in the environment and the Spring Dog should approach the landmark. We compare linear and bilinear dynamics models with quadratic and Gaussian state cost functions. Experimental results showed that the LMDP framework with model learning is applicable to real robot learning even when simple dynamics models are used.

## 2. Materials and methods

### 2.1. Linearly solvable markov decision process

At first, we show how a non-linear Bellman's equation can be made linear under the LMDP setting formulated by Todorov (2009b). Let 
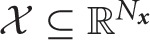
 and 
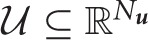
 be the continuous state and continuous action spaces, where *N*_***x***_ and *N*_***u***_ are the dimensionality of the spaces, respectively. At time *t*, the robot observes the environmental current state 
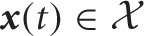
 and executes action 
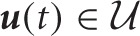
. Consequently, the robot receives an immediate cost *c*(***x***(*t*), ***u***(*t*)) and the environment makes a state transition according to the following continuous-time stochastic differential equation,

(1) d x=a(x)d t+B(x)(ud t+σd ω),

where **ω** ∈ ℝ^*N*_***u***_^ and σ denote Brownian noise and a scaling parameter for the noise, respectively. ***a***(***x***) describes the passive dynamics of the system while ***B***(***x***) represents the input-gain matrix. Note that Equation (1) is generally non-linear with respect to the state ***x*** but linear with respect to the action ***u***. It is convenient to represent Equation (1) in discrete time. By discretizing the time axis with step *h*, we obtain the following transition probability, 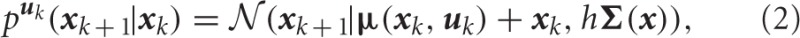 where 
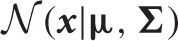
 denotes a Gaussian distribution with mean **μ** and covariance matrix **Σ**, and

(3) μ(x,u)=h(a(x)+B(x)u),

(4) $$\Sigma (x)=\sigma B{\left(x\right)}^{\text{T}}B(x),$$

where **μ**(***x, u***) can be regarded as a deterministic state transition function. Note that ***x***_*k*_ = ***x***(*hk*) and ***u***_*k*_ = ***u***(*hk*). It should be noted that a state transition probability is defined as an uncontrolled probability when no control is applied (***u*** = **0**), and otherwise, it is called a controlled probability.

A control policy or controller π(***u***|***x***) is defined as a probability of selecting the action ***u*** at state ***x***. When the goal of the robot is to find an optimal control policy π^*^ that can lead the robot to the desired state 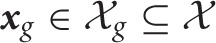, the objective function is formulated as minimization of the expected value of cumulative costs, 
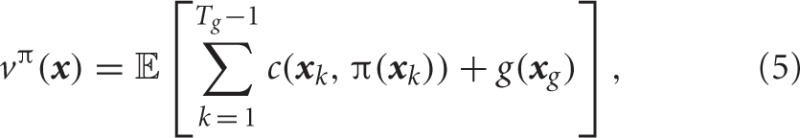
 where and *c*(***x, u***) and *g*(***x***), respectively denote the immediate and terminal cost. *T*_*g*_ represents an arrival time. *v*^π^(***x***) is known as a cost-to-go or value function. The optimal value function is the minimal expected cumulative cost defined by

(6) $$v^{*}(x)=\underset{\text{π}}{\min}v^{\pi }(x).$$

It is known that the optimal value function satisfies the following Bellman's equation 
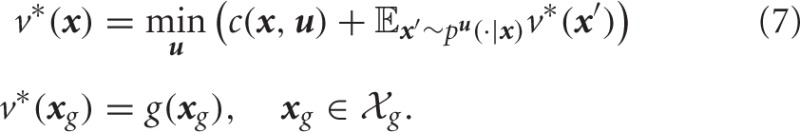
 Since Equation (7) is non-linear, it is difficult to solve the optimal value function in general. However, the Bellman's equation is simplified if it is assumed that the immediate cost function is represented by

(8) $$c(x,u)=hq(x)+\text{KL}(p^{u}(\cdot |x)∥p^{0}(\cdot |x)),$$

where *q*(***x***) is a non-negative state cost function and the second term on the right hand side of Equation (8) is a control cost given as the KL-divergence between controlled and uncontrolled probability distributions.^1^ In this case, the non-linear Bellman's equation is converted to the following linear equation 
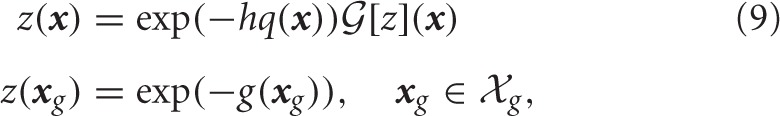
 where *z*(***x***) is the desirability function defined by

(10) $$z(x)=\exp(-v^{*}(x)).$$

Hereafter, Equation (9) is called a linearized Bellman's equation. The operator 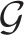 shown on the right hand side of the linearized Bellman's Equation (9) is the integral operator given by 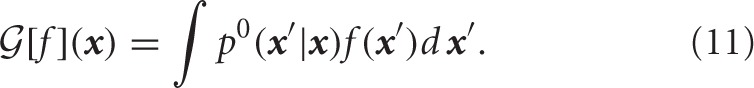 It should be noted that Equation (9) is always satisfied by the trivial solution (*z*(***x***) ≡ 0 for all ***x***) if no boundary conditions are introduced.

### 2.2. Learning model parameters

In the LMDP framework, the system dynamics (Equation 1) are assumed to be known in advance. When they are unknown, estimation of the dynamics is required from samples collected by the passive dynamics. Many methods exist which can estimate the system dynamics (Nguyen-Tuong and Peters, 2011; Sigaud et al., 2011), we adopt a simple least squares method to estimate ***a***(***x***) and ***B***(***x***) with basis functions. Specifically, we estimate a deterministic state transition (Equation 3). It should be noted that the scale parameter of noise σ is generally unknown, but it is determined by the experimenters here since it can be regarded as the parameter that controls exploration of the environment.

Let us suppose that the deterministic state transition **μ**(***x, u***) is approximated by the linear function with *N*_φ_ basis functions φ_*i*_(***x, u***),

(12) $$\mu (x,u;W)=W^{\text{T}}\varphi (x,u).$$

where ***W*** is a weight matrix and **φ**(***x, u***) is a vector consisting of basis functions. Suppose that the training samples {***x***_1_, ***u***_1_, …, ***x***_*N*_*s*__, ***u***_*N*_*s*__, ***x***_*N*_*s*+1__} are obtained by the passive dynamics. The objective function of model learning is given by the following sum-of-squares error function,

(13) $$E=\frac{1}{2}\sum_{k=1}{\left\{\Delta x_{k}-W^{\text{T}}\varphi (x_{k},u_{k})\right\}}^{2},$$

where Δ***x***_*k*_ = ***x***_*k* + 1_ − ***x***_*k*_. Setting ∂*E*/∂***W*** = **0** yields

(14) $$W={\left(\Phi^{\text{T}}\Phi \right)}^{-1}\Phi^{\text{T}}\Delta X,$$

where Δ***X*** is the matrix whose a row vector consisted of state transition in each sample Δ***x***_*k*_ and **Φ** is also the matrix whose a column vector consisted of the basis functions in each sample **φ**(***x***_*k*_, ***u***_*k*_). The detail is as follow,

$$\Delta X={\left[\begin{array}{ccc} \Delta x_{1} & \cdots & \Delta x_{N_{s}} \end{array}\right]}^{\text{T}},\;\Phi =\left[\begin{array}{ccc} \varphi (x_{1},u_{1}) & \cdots & \varphi (x_{N_{s}},u_{N_{s}}) \end{array}\right].$$

### 2.3. Learning a desirability function

The desirability function is approximated by

(15) $$z(x;w,\theta )=\sum_{i=1}^{N_{z}}w_{i}f(x,\theta_{i})=w^{⊤}f(x,\theta ),\;$$

where *w*_*i*_ is a weight, ***w*** is the weight vector [*w*_1_, …, *w*_*N*_*z*__]^T^, *f*(***x***, **θ**_*i*_) is a basis function parameterized by **θ**_*i*_, and ***f***(***x***; **θ**) is the vector consisting of basis functions [*f*(***x***; θ_1_), …, *f*(***x***; θ_*N*_*z*__)]^T^. We adopt an unnormalized Gaussian function as Todorov suggested (Todorov, 2009c):

(16) $$f(x;\theta_{i})=\exp\text{​}\left(-\frac{1}{2}{\left(x-m_{i}\right)}^{\text{T}}S_{i}\left(x-m_{i}\right)\right),\;\theta_{i}=\{m_{i},S_{i}\}$$

where ***m***_*i*_ and ***S***_*i*_ denote a center position and a precision matrix of the *i*-th basis function, respectively. One advantage of using the Gaussian function that the integral operator (Equation 11) can be calculated analytically as follows: 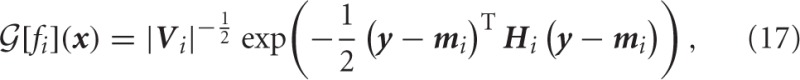 where ***y***(***x***) = ***x*** + **μ**(***x***, **0**), *f*_*i*_ = *f*(***x***, **θ**_*i*_) for brevity and

$$\begin{array}{l} H_{i}=S_{i}-S_{i}CV_{i}^{-1}C^{\text{T}}S_{i},\;V_{i}=I+C^{\text{T}}S_{i}C,\\ \;C=\sigma h^{1/2}B. \end{array}$$

It should be noted that ***y, H***_*i*_, ***V***_*i*_, ***C*** are functions of ***x***.

The desirability function (Equation 15) should satisfy the linearized Bellman's equation (9). Therefore, in order to optimize ***w*** and **θ** we can construct the following objective function for given collocation states {***x***_1_, …, ***x***_*N*_*c*__}:

(18) $$e=\|r(w,\theta ){\|}^{2},\;r(w,\theta )=\left[\begin{array}{c} F(\theta )-G(\theta )\\ f{\left(x_{g};\theta \right)}^{\text{T}} \end{array}\right]w-\left[\begin{array}{c} 0\\ \exp(-g(x_{g})) \end{array}\right],$$

where ***F***(**θ**) and ***G***(**θ**) are *N*_*c*_ × *N*_*z*_ matrices and their (*n, i*) components are defined by 
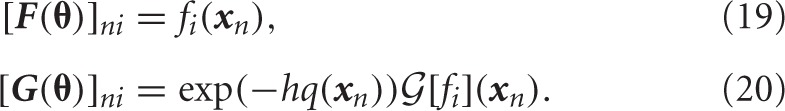
 The objective function (Equation 18) is a quadratic function with respect to ***w*** and a non-linear function with respect to **θ**. See Appendix for optimization of ***w*** and **θ**.

### 2.4. Optimal control policy

In the LMDP framework, the optimal control policy is given by 
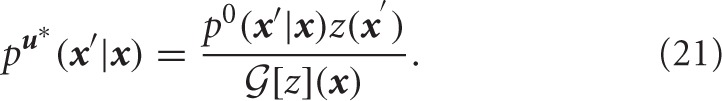
 Specifically, when the dynamics are represented in the form of the stochastic differential equation (1) and the basis function of the approximated desirability function is Gaussian, then the optimal control policy is represented by 
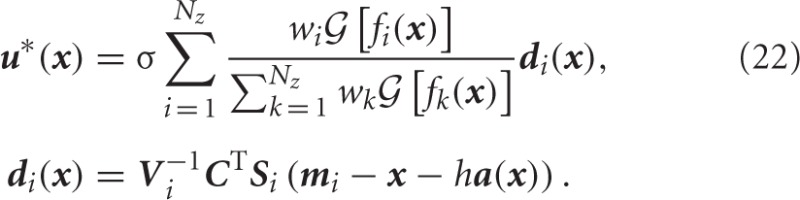
 See Todorov (2009c) in more detail.

### 2.5. Experiment

In this paper, we conduct two experiments to evaluate the LMDP framework with model learning. One is a swing-up pole task in simulation. The other is a visually-guided navigation task using a real robot.

#### 2.5.1. Swing-up pole

**Figure 1:**
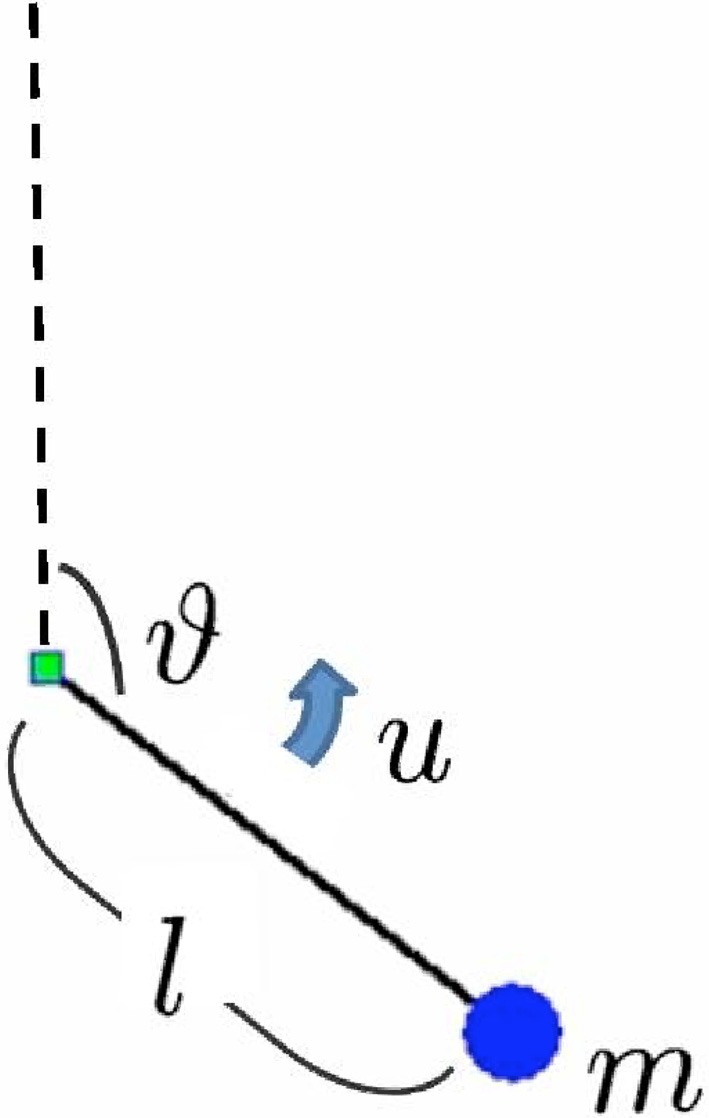
**Swing-up pole task**.

To verify that an appropriate control policy can be derived based on estimated dynamics, we conducted a computer simulation of the swing-up pole task. In the simulation, the one side of pole was fixed and the pole could rotate in plane around the fixed point as shown in Figure 1. The goal was to swing the pole to an upward position and stop at this position. The state in this task consisted of the vertical angle ϑ and the angular velocity $\dot{\vartheta }$, the origin of the state space was set at the goal position. It should be noted that ϑ was normalized to be in the range (−π, π] (rad) while $\dot{\vartheta }$ was bounded: $\dot{\vartheta }\in [-4\pi ,\;4\pi ]$ (rad /s). The control input and noise affected the torque of the pole. Therefore, the pole is assumed to obey the following stochastic state equation,

(23) $$\begin{array}{l} d\vartheta =\dot{\vartheta }dt\\ d\dot{\vartheta }=\left(m\frac{g}{l}\sin(\vartheta )-k\dot{\vartheta }\right)dt+udt+\sigma d\omega , \end{array}$$

where *l*, *m*, *g*, and *k* denote the length of the pole, mass, gravitational acceleration and coefficient of friction, respectively. The above state equation is represented in the form of Equation (1) as follows;

$$a(x)={\left[\begin{array}{c} \dot{\vartheta },\;m\frac{g}{l}\sin(\vartheta )-k\hat{\vartheta } \end{array}\right]}^{\text{T}},\;B={\left[\begin{array}{c} 0,\;1 \end{array}\right]}^{\text{T}}.$$

It should be note that the passive dynamics ***a***(***x***) is a non-linear vector function of ***x*** while ***B*** is a constant vector. In this simulation, the physical parameters were *l* = 1 (m), *m* = 1 (kg), *g* = 9.8 (kg/s^2^) and *k* = 0.05 (kg m^2^/s). The state equation was discretized in time with a time step of *h* = 10 (ms) and the noise scale was set to σ = 4. The state cost was defined so that it was zero at the goal state, using the following unnormalized Gaussian function,

(24) $$q(x)=\left(1-\exp\left(x^{\text{T}}\Sigma_{\text{cost}}^{-1}x\right)\right),$$

where diag (Σ_cost_) = [0.1, 1.6].

As written in section 2.2, the weight matrix was estimated by Equation (14). In the sample acquisition phase we repeated simulations sufficiently, each simulation started from different initial states to avoid unevenly distributed samples. As a result, *N* = 1000 samples were extracted randomly as a training data set.

In this simulation, we prepared two types of basis functions **φ**(***x, u***), as shown in Table 1, for approximation of the environmental dynamics. The first was a simple linear model with respect to ***x*** and ***u*** while the second model added the normalized radial basis functions (NRBF) ψ_*i*_(***x, u***) to the linear model,

(25) $$\psi_{i}(x)=\frac{\exp\text{​}\left(-\frac{1}{2}{\left(x-\mu_{i}\right)}^{\text{T}}\Sigma_{\psi_{i}}^{-1}(x-\mu_{i})\right)}{\sum_{k}\exp\text{​}\left(-\frac{1}{2}{\left(x-\mu_{k}\right)}^{\text{T}}\Sigma_{\psi_{k}}^{-1}(x-\mu_{k})\right)}.$$

**Table 1:** **Basis functions used in the swing-up pole simulation**.

	**φ**(***x, u***)
Linear model	[***x***^⊤^ ***u***^⊤^]^⊤^
Linear-NRBF model	[***x***^⊤^ ψ_1_(***x***) ψ_2_(***x***) … ψ_*M*_(***x***) ***u***^⊤^]^⊤^

The centers, **μ**_*i*_, of the basis functions, ψ_*i*_(***x, u***), were determined by *K*-means clustering among the states of the training data. The covariance matrices **Σ**_ψ_*i*__ were determined experimentally and set to diag(**Σ**_ψ_*i*__) = [π/4, π]. In the linear-NRBF model, *N*_ψ_ = 25 basis functions were used.

The set of collocation states {***x***_1_, …, ***x***_*N*_*s*__}, which were required to optimize the parameters of the desirability function, were uniformly distributed in the state space. The centers ***m***_*i*_ of the basis functions *f*_*i*_(***x***) were initialized so as to distribute them uniformly in the state space. On the other hand, the covariance matrices ***S***_*i*_ were determined empirically and set to diag([16, 1]). The optimal control policy u^*^(***x***) was derived from Equation (22).

#### 2.5.2. Visually-guided navigation task

**Figure 2:**
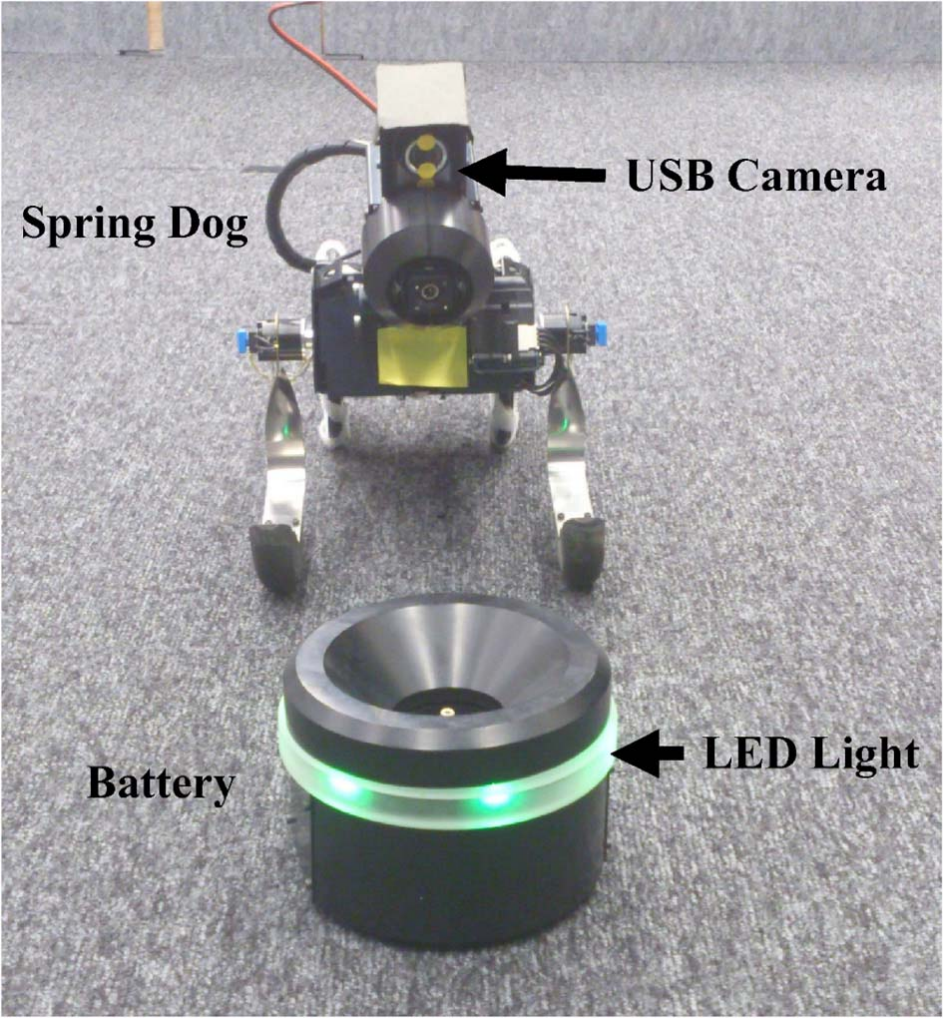
**Spring Dog, wheel typed robot and the battery pack**.

To evaluate the performance of the optimal control policy derived from the estimated dynamics and the desirability function, we conducted a visual navigation task using a wheel type robot called the Spring Dog. Figure 2 shows the Spring Dog and the battery pack in the experimental field. The Spring Dog has six degrees of freedom: two fore legs, two rear wheels, and a pan-tilt camera head. There are several sensors such as a 3D accelerometer, a combined 3D gyroscope, and a USB camera mounted on the head, and so on. Three-color LED is attached to the top of the battery pack.

**Figure 3:**
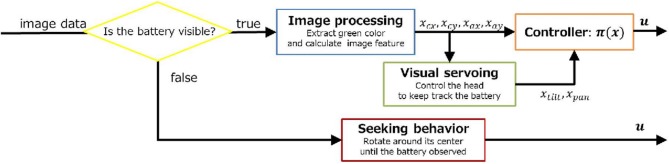
**Control diagram in the Spring Dog**.

Figure 3 shows the control diagram, where three control policies were implemented in this experiment. The first one was a visual servoing controller, which controlled the camera head so as to keep tracking the battery pack continuously. The second one was a navigation controller using the two rear wheels, this was optimized by the LMDP framework. In other words, the navigation controller controlled the left and right wheels in order to move around in the environment. The desired velocities of left and right wheels correspond to control input ***u*** in Equation (1). The last one was a seeking behavior, in which the Spring Dog explored the environment to find the battery pack when the robot lost track of it. The navigation controller learned by the LMDP framework while the visual servoing and searching controllers were designed by the experimenters.

**Figure 4:**
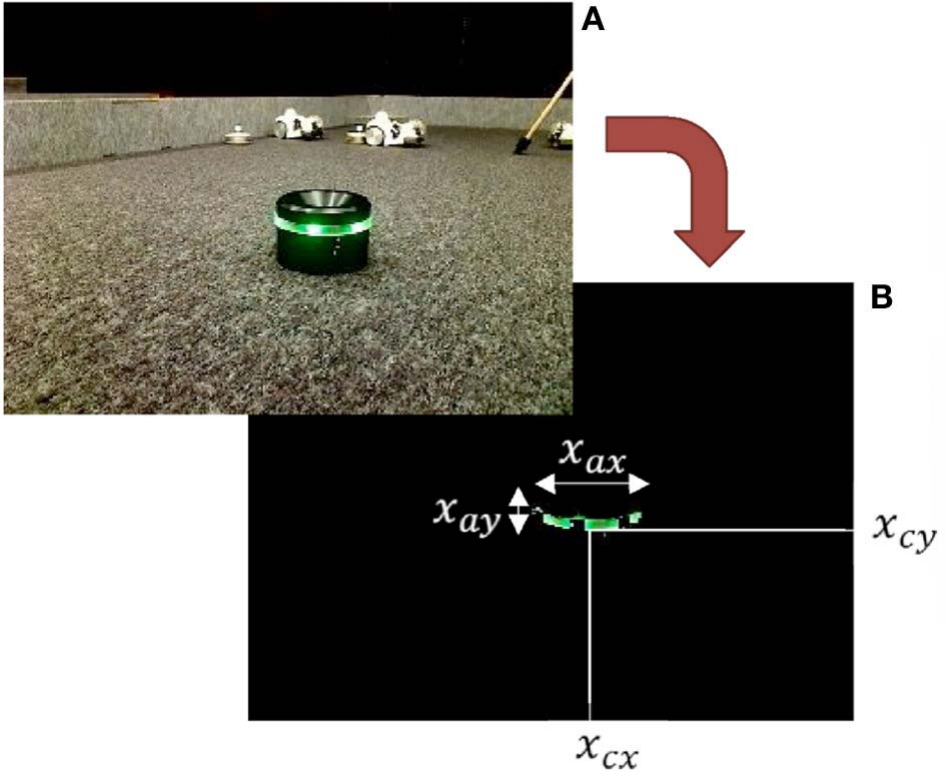
**Image binarization and image features. (A)** Original captured image. **(B)** Binarized image.

To realize a visually-guided navigation task, image binarization was applied to a captured image in order to separate the battery pack with the green LED from background. Some image features were calculated as shown in Figure 4. The state space consists of six variables described below: the center position of the battery pack (extracted pixels) in the image plane (*x*_*cx*_, *x*_*cy*_), average of absolute values around the center in horizontal and vertical axes of the extracted pixels (*x*_*ax*_, *x*_*ay*_), and the current joint angles of the neck controlled by the visual servoing controller. The state and action were summarized as follows:

$$x={\left[x_{cx},x_{cy},x_{ax},x_{ay},x_{\text{tilt}},x_{\text{pan}}\right]}^{\text{T}},\;u={\left[u_{\text{left}},u_{\text{right}}\right]}^{\text{T}}.$$

It should be noted that each value was scaled as follow,

$$\begin{array}{l} -1\leq \;x_{cx},x_{cy},x_{\text{tilt}},x_{\text{pan}}\leq 1,\\ \;0\leq \;x_{ax},x_{ay}\leq 1,\\ -1\leq \;u_{\text{left}},u_{\text{right}}\leq 1. \end{array}$$

The desired state, ***x***_*g*_, was set to comprise of both a posture and location which allowed the Spring Dog to successfully capture of the battery. The view feed from the USB camera allowed recognition of the desired proximity and posture, as shown in Figure 2.

Two types of state dependent cost functions *q*_1_(***x***) and *q*_2_(***x***) were considered in the experiment. Each cost function was defined to be zero at the goal state as follows,

(26) $$q_{1}(x)=\alpha {\left(x-x_{g}\right)}^{\text{T}}\Sigma_{\text{cost}}^{-1}\left(x-x_{g}\right)$$

(27) $$q_{2}(x)=\alpha \left(1-\exp\text{​}\left(-{\left(x-x_{g}\right)}^{\text{T}}\Sigma_{\text{cost}}^{-1}\left(x-x_{g}\right)\right)\right),$$

where α was a scaling constant.

Next we explain the procedure for estimation of visual-motor dynamics. At first, the Spring Dog moved around using the fixed stochastic policy and obtained data. In the experiment, the control cycle was required to keep *h* = 300 ± 60 (ms), but it was sometimes violated interference from other processes. To deal with this problem in sampling, we rejected the corresponding data. In addition, If the target became invisible, or the tilt or pan angle reached by setting, its limitation, the corresponding data was rejected from samples also. As a result, we obtained the data set, 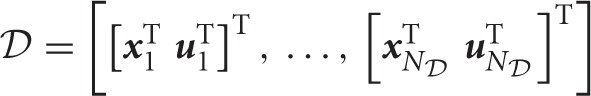. After normalizing this data set, the environmental dynamics were estimated as described in section 2.2.

In this experiment, we used two types of basis functions **φ**(***x, u***), as shown in Table 2, to estimate visual-motor dynamics. If we apply the linear model for visual-motor dynamics and use a quadratic state cost function in Equation (26), the problem setting is identical to that of Linear Quadratic Regulator (LQR). Therefore, we can confirm that the LMDP finds the same optimal policy as LQR.

**Table 2:** **Basis functions used in the robot experiment**.

	**φ**(***x, u***)
Linear model	[(***x*** − ***x***_*g*_)^⊤^ ***u***^⊤^]^⊤^
Bilinear model	[(***x*** − ***x***_*g*_)^⊤^ *u*_left_(***x*** − ***x***_*g*_)^⊤^ *u*_right_(***x*** − ***x***_*g*_)^⊤^ ***u***^⊤^]^⊤^

As well as the swing-up pole task, collocation states {***x***_1_, …, ***x***_*N*_*s*__} were uniformly distributed in the state space, and the covariance matrices ***S***_*i*_ were determined by hand. Moreover, only centers of basis functions of desirability were updated and covariance matrices were fixed in the experiment. The optimal control policy u^*^(***x***) was derived from Equation (22). The initial position of the center ***m***_*i*_ in each basis function *f*_*i*_(***x***) was taken from the data set of state, 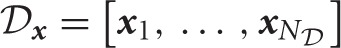, which was extracted state data from the data set 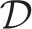. However, it was not appropriate for the computational resources of the real robot to use all of the data. For this reason, the set of initial positions of the centers of the basis functions, **M**_*init*_ = [***m***_1_, …, ***m***_*N*_*z*__], were chosen from the data set of state 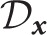 following Procedure 1. As a result, at least one of the basis functions could return the value, which was over the threshold, τ, for every samples.


**Procedure 1 | Setting initial position of the centers of the basis functions, M_*init*_.**
____________________________________________________________________________________
**Input**: The date set of state, ***D***_*x*_.
**Output**: The set of initial center positions, **M**_*init*_
   **M**_*init*_ ← ∅
   while ***D***_*x*_ ≠ ∅ **do**
     *x* = ChooseSample(***D***_*x*_)
     ***D***_*x*_ ← **X**_***D***_ − {*x*}
     **if** ∀*i* *f*_*i*_(*x*; *m*_*i*_) < τ or **M**_*init*_ = ∅ **then**
        **M**_*init*_ ← **M**_*init*_ ∪{*x*}
     **end if**
     **end while**
     **return M**_*init*_


As already explained, to verify that LMDP can be apply to non-linear state transition system and non-quadratic cost function and the obtained controller performs optimal. In the experiment we tested the following four conditions:

1. Linear model + quadratic state cost.
2. Bilinear model + quadratic state cost.
3. Linear model + Gaussian based state cost (non-quadratic).
4. Bilinear model + Gaussian based state cost (non-quadratic).

Note that LQR can be applicable in the first condition. Therefore, LQR was also implemented to compare the result of the LMDP framework to the ground truth obtained from LQR in the first condition.

## 3. Results

### 3.1. Computer simulation

As described in the section 2.5.1, we used the linear and the linear-NRBF models to approximate the environmental dynamics of the swing-up pole. To evaluate the accuracy of estimation using these models, we measured the estimation errors. We extracted *N* = 500 samples randomly as a test data set and then calculated the estimates of the deterministic state transition **μ**(***x, u***; ***W***) when two models were applied, respectively. After that, we computed the mean squared error (MSE) of each component,

(28) $$\text{MSE of the }k\text{-th component}=\frac{1}{\text{N}}\sum_{\text{n=1}}^{\text{N}}{\left(\Delta {\text{xk}}_{{}_{\text{n}}}\text{-}w_{\text{k}}\varphi (x_{\text{n}},u_{\text{n}})\right)}^{\text{2}},$$

where ***w***_*k*_ denotes the elements of *k*-th row in the weight matrix ***W***.

**Figure 5:**
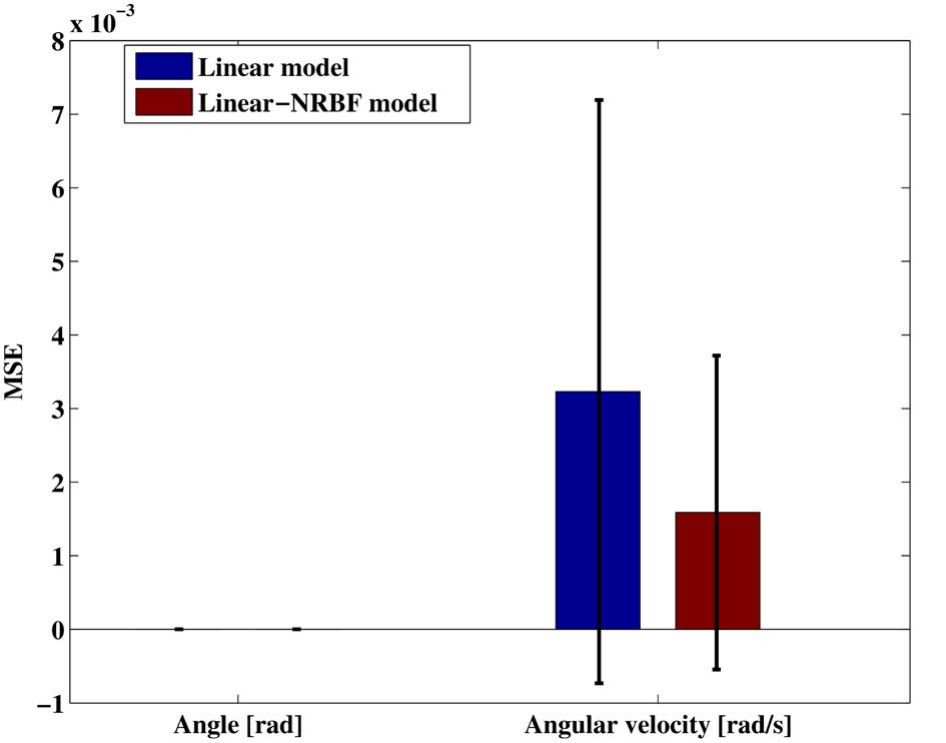
**Mean squared error of the joint angle and angular velocity.** Each error bar represents the standard deviation.

Figure 5 shows the MSE of the angle and angular velocity component. According to the this result, the estimation of the angle component was quite accurate in both models because it was deterministic transition. On the other hand, the estimation of the angular velocity component was inaccurate as compared with the angle component since it was a stochastic state transition. According to Equations 2, 3 and the parameter setting of the time step, *h* = 10 (ms), the noise scale, σ = 4, and **B** = [0, 1]^T^, the covariance matrix was derived diag (Σ) = [0, 0.04]. The covariance matrix affects to the MSE by square, the MSE between real deterministic state transition and an observed temporal state transition should be at least 1.6 × 10^−3^. The MSE of angular velocity component in the linear-NRBF model was also 1.6 × 10^−3^, it was suggested that most of the error was caused by noise. Consequently, This result suggested that the environmental dynamics were accurately approximated by the linear-NRBF model. The estimated input gain matrices were given by

$$B_{\text{linear}}=\left[\begin{array}{c} 0.0000\\ 0.9965 \end{array}\right],\;B_{\text{linear-NRBF}}=\left[\begin{array}{c} 0.0000\\ 1.0113 \end{array}\right].$$

they were very close to the true matrix ***B*** = [0, 1]^T^.

**Figure 6:**
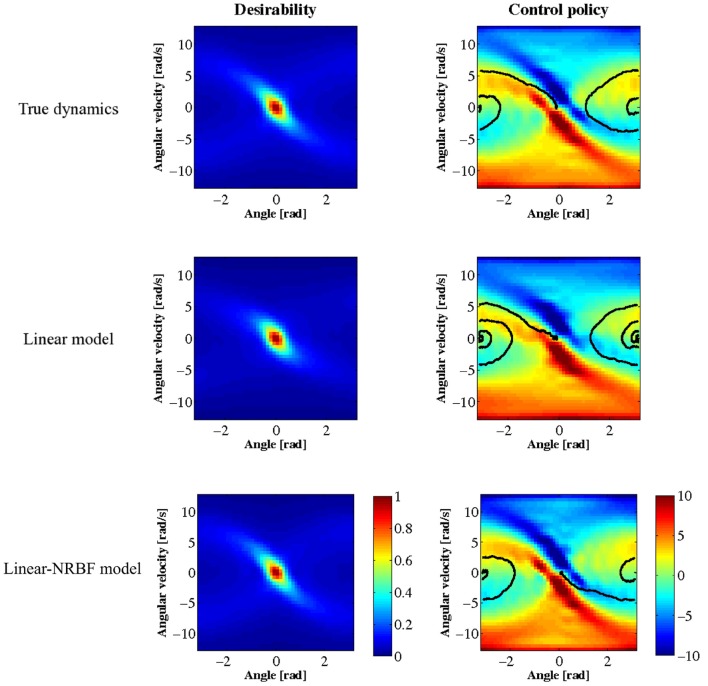
**Results of the swing-up pole task.** *z*(***x***) is on the left, u^*^(***x***) on the right, true dynamics at the top, linear model in the middle, and linear and NRBF model at the bottom. The black line shows a typical trajectory.

The desirability function was optimized using the estimated dynamics and the control policy derived from the obtained desirability function. Figure 6 displays the results where the left panels show the desirability function *z*(***x***) and the right panels show the learned policy u^*^(***x***). The black line in the right panels shows a typical trajectory of learned behaviors starting from ***x*** = [π, 0]^T^. The top panels of Figure 6 display the results using the true dynamics. It should be noted that the desirability function is discontinuous around the central diagonal band since this system is under-actuated. Simulation results using the linear and linear-NRBF models are shown in the middle and bottom panels of Figure 6, respectively. As compared with the result based on the true dynamics, both of the linear and linear-NRBF models could approximate the desirability function. However, the policy obtained by the linear model was worse than that by the linear-NRBF model.

**Figure 7:**
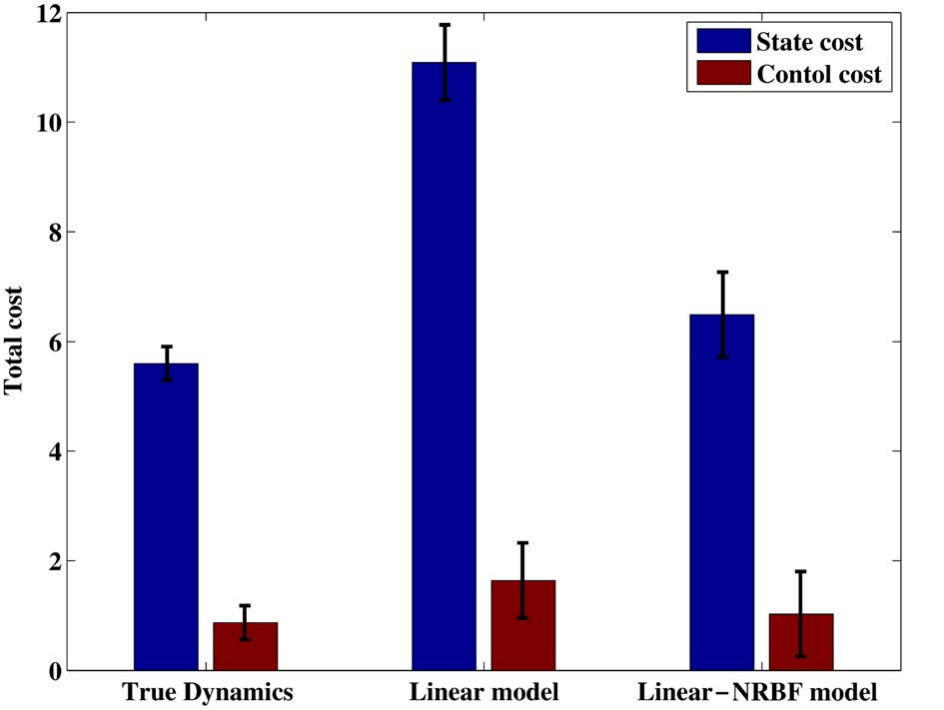
**Total costs collected by the obtained control policy.** Each error bar represents the standard deviation.

To evaluate the performance in more detail, we measured the cumulative costs corresponding to each of the obtained policies. In this test simulation, the initial state was set to ***x*** = [π, 0]^T^ which corresponds to the bottom position. Figure 7 shows mean cumulative costs of 50 episodes, each episode was terminated when the pole arrived at the goal state or the duration reached was over 20 (s) (2000 step). Note that the immediate cost in each step was calculated by $c(x,u)=h\left(q(x)+\frac{1}{2\sigma^{2}}\|u{\|}^{2}\right)$.

Figure 7 compares the cumulative costs among the three policies. Not surprisingly, the control policy derived from the true dynamics achieved the best performance. It should be noted that the control policy based on the dynamics estimated with the linear-NRBF model produced a comparable performance, and it was better than the performance of the linear model. As discussed in the previous section, the linear-NRBF model gave more correct estimation than the linear model. Consequently, these results suggest that we can obtain the better control policy by forming more accurate estimates.

### 3.2. Real robot experiment

As described in section 2.5.2, we used the linear and bilinear models for environmental dynamics approximation. After the data acquisition phase, we obtained 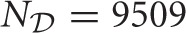 samples and we extracted *N* = 2500 samples for a test data set, the rest of samples were used as a training data set. As well as the swing-up the pole task, we obtained weight matrix using Equation (14) and then calculated MSE in the test data set to evaluate the accuracy of estimation.

**Figure 8:**
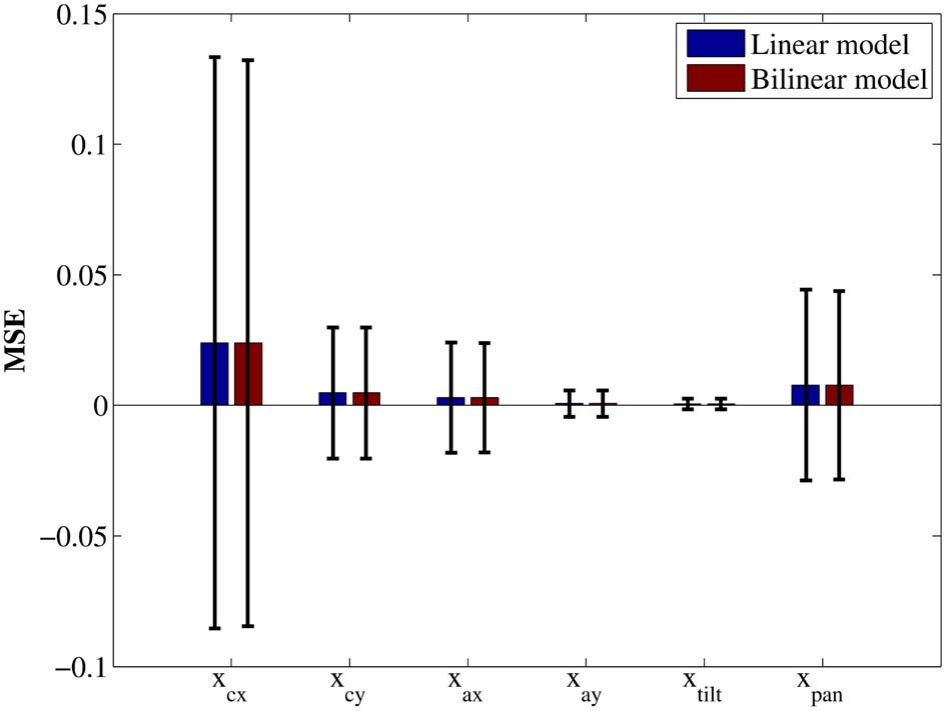
**Mean squared error of each state variable.** Each error bar represents the standard deviation.

Figure 8 shows the result. There was no significant difference between linear and bilinilear models. It suggests these models have almost the same quality for approximating environmental dynamics. Comparing to other components, *x*_*cx*_ and *x*_pan_ derive larger MSE in both model. The reason is these components change more significantly than other components. During the sample acquisition phase, more movement in the rotatory direction occurred than in the translation direction. As a result, the variation of *x*_*cx*_, which was caused by movement of rotatory direction, was large and the variation of *x*_pan_ also became large due to visual servoing to keep track of the battery in center of visual field.

**Figure 9:**
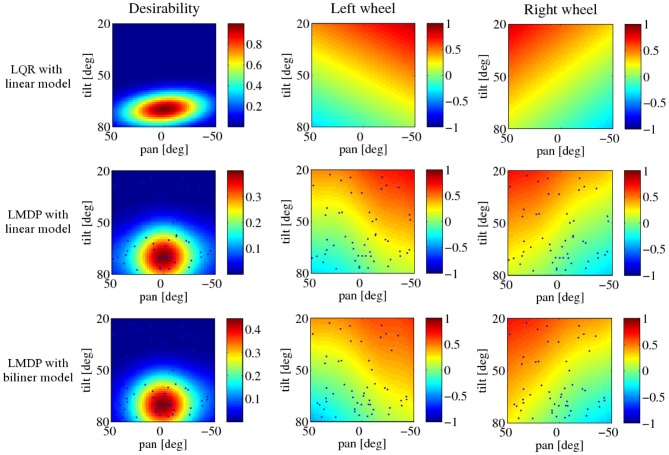
**Results of the robot navigation task.** LQR with the linear model is at the top, LMDP with the linear model in the middle, LMDP with the bilinear model at the bottom, *z*(***x***) on the left, u^*^_left_(***x***) on the center, u^*^_right_(***x***) on the right. Black dots represent the centers of the basis functions **φ**(***x, u***).

Figure 9 shows one typical example of the obtained desirability function and the control policy when the cost function is quadratic and the visual-dynamics is estimated using the linear and bilinear models. The upper row corresponds to the LQR's case and the middle and bottom rows correspond to the LMDP trained with the proposed method using linear and bilinear models, respectively. In all figures, the horizontal and the vertical axes denote the pan and tile angle of the neck joint, respectively; the rest of the state components are set to the desired state. Blue dots plotted on middle and lower rows are ***m***_*i*_, the center positions of the basis functions for approximating the desirability function. Although the peak of the desirability functions trained with the proposed method is broader than that of the desirability of LQR due to function approximation, obtained controllers show almost same tendency.

**Figure 10:**
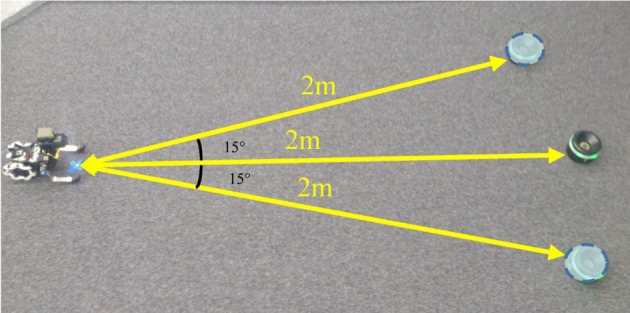
**Initial position of the Spring Dog and battery in the test phase.** Three possible positions of the battery pack are considered.

**Figure 11:**
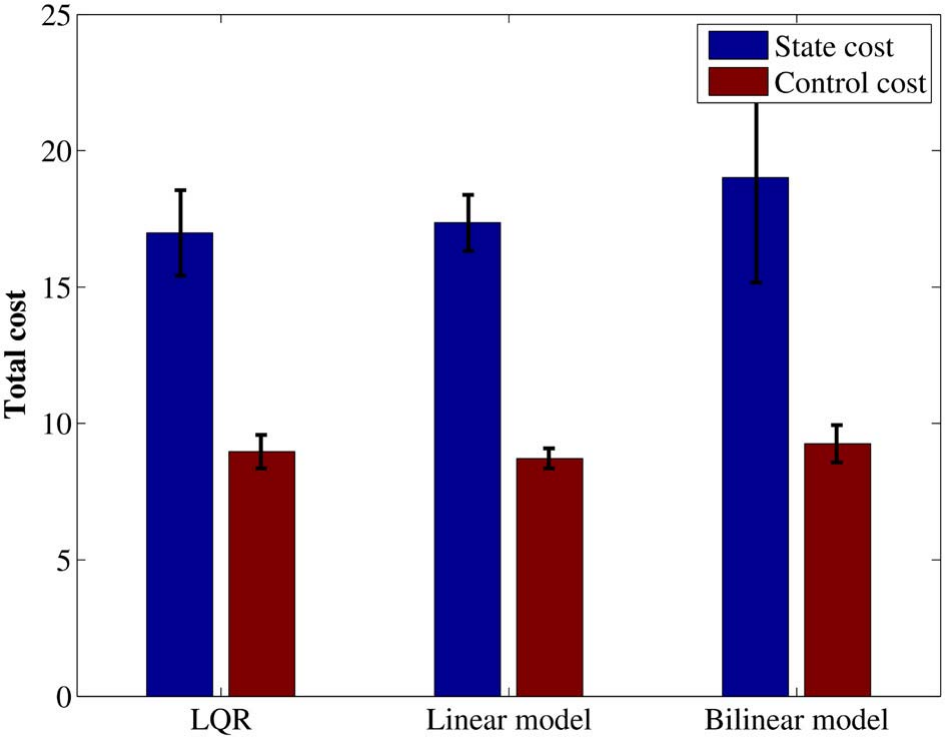
**Average of total cost using the quadratic state cost function.** Each error bar represents the standard deviation.

Next, to evaluate performance of obtained controllers, we tested the approaching behavior under the each controller. In the test, the initial position of the robot was set at a distance of 1.5 (m) left the target. The initial direction for each episode was selected randomly a set of three directions; target is placed directly in front of the robot, at a 15° offset to the right of the robot's line of motion or at a 15° offset to the left side, as shown in Figure 10. Figure 11 shows the mean total costs of 30 episodes, the maximum period in one learning episode was 15 (s) (50 steps). For comparison, Figure 11 shows only quadratic cost function case. Note that the immediate cost in each step was regarded as $c(x,u)=h\left(q_{1}(x)+\frac{1}{2\sigma^{2}}\|u{\|}^{2}\right)$, and was ignored when the target is not visible in the visual field.

**Figure 12:**
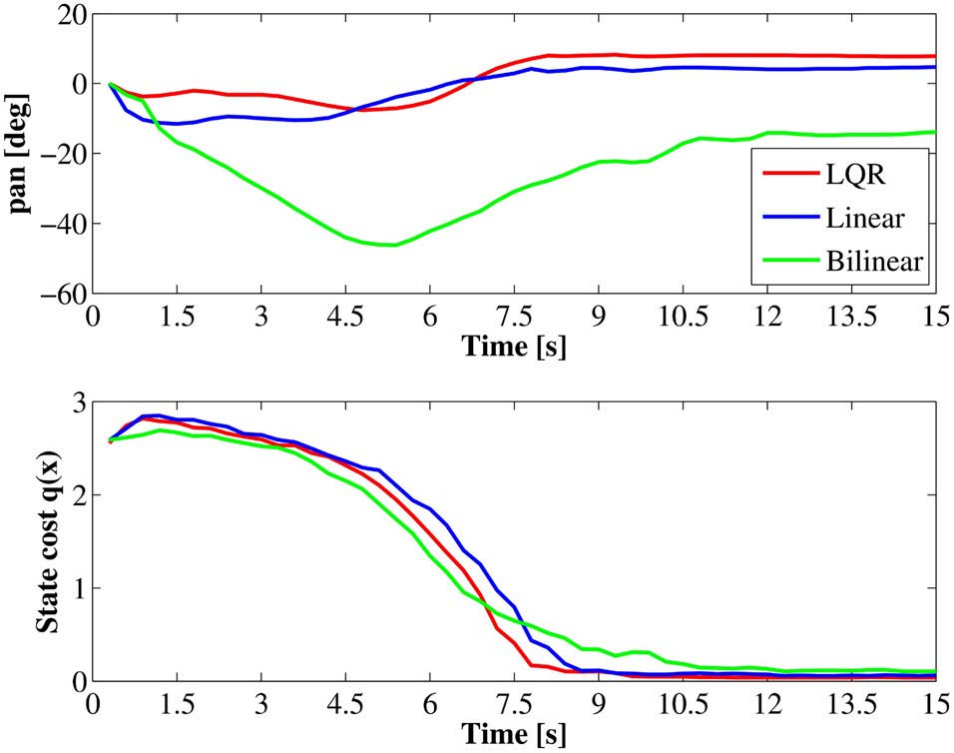
**Trajectories of the pan angle *x*_tilt_ and the immediate state cost under the quadratic state cost**.

Comparing the total cost among the three controllers using quadratic cost as shown in Figure 11, the controller using the linear model resulted in the almost same performance to the result using LQR controller. This result is reasonable because these controllers solve the same problem. The trajectories were very similar shown in Figure 12.

On the other hand, the controller using a bilinear model acquired marginally worse result as compared with the other controllers. One possible reason is that over fitting occurred in bilinear model.

**Figure 13:**
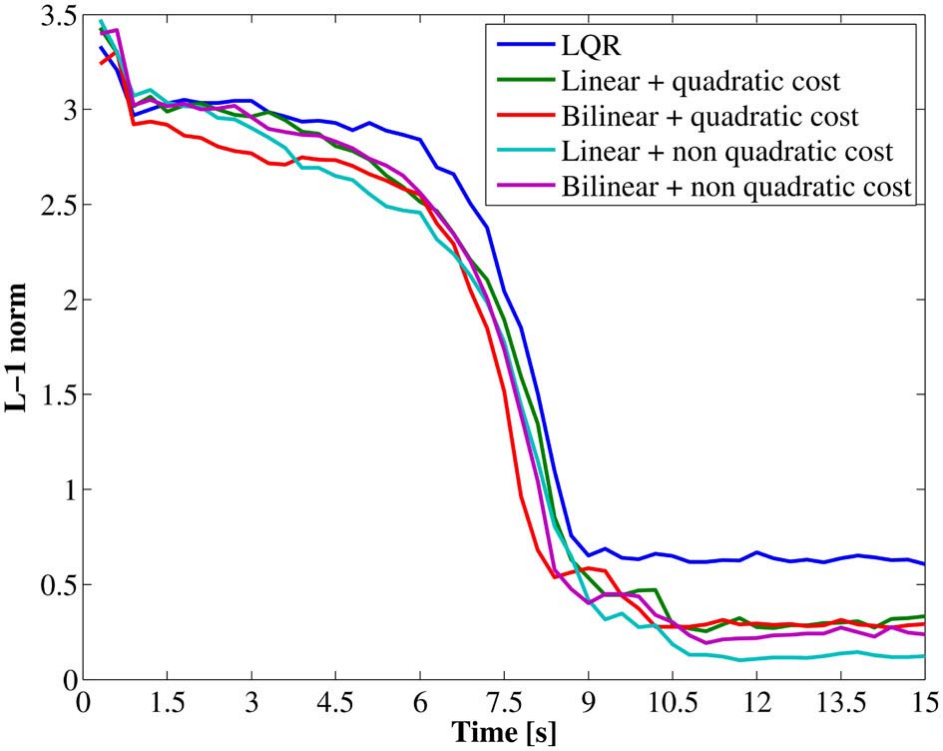
**Trajectories of the L-1 norm between the current and goal states**.

In comparing performance among all obtained controllers, we cannot use the total cost because of the difference on state costs. For this reasons we calculated L-1 norm^2^ between the current state and the goal state as quantity of controller performance which can be comparable in all controllers. Figure 13 shows this. All of controllers brought the Spring Dog to almost the goal state in 10 s. Particularly, the controllers using the non-quadratic cost function brought the Spring Dog closer to the battery pack than other controllers. The reason can be considered that the non-quadratic cost function gave a lower cost in more narrow region than the quadratic cost.

## 4. Discussion

Although it has been reported that the framework of LMDP can find an optimal policy faster than conventional reinforcement learning algorithms, the LMDP requires the knowledge of state transition probabilities in advance. In this paper, we demonstrated that the LMDP framework can be successfully used with the environmental dynamics estimated by model learning. In addition, our study is the first attempt to apply the LMDP framework to real robot tasks. Our method can be regarded as a of model-based reinforcement learning algorithms. Although many model-based methods includes model learning (Deisenroth et al., 2009; Hester et al., 2010) have been proposed in this field, they compute an optimal state value function which is a solution of a non-linear Bellman's equation. Experimental results show that our method is applicable to real robot behavior learning which is generally stochastic and including non-linear state transition. In our proposed method, a cost function is not estimated. However, it is possible to extend to estimate a cost function as well as system dynamics simultaneously, because it is usually formulated as a standard supervised learning problem. In addition, it is not so difficult to assume that a cost function is given in the real robot application, because the robot usually compute the reward by itself in many application.

In the swing-up pole task, the linear and linear-NRBF models were tested to approximate the pole dynamics. The policy derived from the linear model achieved the task of bringing the pole to the desired position even though it cannot represent the dynamics correctly. In the visually-guided navigation task, we compared the desirability function and control policy of LMDP with those of LQR if the environmental dynamics and the cost function were approximated by the linear model and the quadratic function, respectively. In this setting, the optimal state value function and the control policy were calculated analytically by LQR, and therefore, we obtained the optimal desirability function. The obtained desirability function and control policy were not exactly the same as those of LQR. However, we confirmed that the performance using the obtained control policy was comparable to the performance using LQR. Both models prepared in this experiment failed to approximate a part of state transition such as *x*_*cx*_ and *x*_pan_. This means that the Spring Dog could not predict the future position of the battery pack precisely when turned left or right. Nevertheless, the robot could approach the battery pack appropriately. This result suggests that LMDP with model learning is promising even though the estimated model was not so accurate. Fortunately, the control policy which brings the robot to the desired position can be obtained with simple linear model in both experiments. We plan to evaluate the proposed method to non-linear control tasks such as learning walking and running behaviors.

As discussed in section 3, the quality of obtained control policy depends on the accuracy of the estimated environmental model. For instance, the bilinear model used in the robot experiment did not improve the approximation accuracy, as shown in Figure 8, even though its computational complexity is a rather than the linear model. In addition, a part of the conditional mean **μ**(***x, u***) was estimated by the least squares method in the current implementation but it would be more informative to estimate the state transition probability distribution *p*^***u***_*k*_^(***x***_*k*+1_|***x***_*k*_) itself. There exist several methods for estimating a probability distribution from samples. For example, Gaussian process is widely used to estimate environmental dynamics (Deisenroth et al., 2009; Deisenroth and Rasmussen, 2011). Sugiyama et al. (2010) proposed the method to estimate a conditional density distribution efficiently in the manner of density ratio estimation and applied it to state transition estimation in simulated environments. One advantage of their method is that it can estimate a multi-modal distribution by the least squares method. In this case, it is no longer tractable analytically to compute the integral operator even if Gaussian basis functions are used for approximation, and it should be replaced by the Monte Carlo estimates. Integration of sophisticated model learning methods with the LMDP framework is our future work.

The other extension is to develop a model free approach of learning desirability functions, in which the environmental dynamics is not estimated explicitly. Z learning is a typical model-free reinforcement learning method which can learn a desirability function for discrete states and actions, and it was shown that the learning speed of Z learning was faster than that of Q-learning in grid-world maze problems (Todorov, 2007, 2009b). Application of least squares-based reinforcement learning algorithms (Boyan, 2002; Lagoudakis and Parr, 2003) is promising direction. However, in the continuous state case, as mentioned in section 2.1, the optimality equation derive a trivial solution without boundary conditions. In addition, the desirability function should satisfy the inequality 0 ≤ *z*(***x***) ≤ 1 in order to recover a correct value function by *v*(***x***) = −log(*z*(***x***)). Furthermore, values of the desirability “function tend to be too small” because of the exponential transformation. For these reasons boundary conditions must be carefully considered. Consequently, the constrained optimization methods should be solved to find the optimal desirability function while learning of the value function is considered as unconstrained optimization. For the extension of model-free learning, this issue have to be solved.

### Conflict of interest statement

The authors declare that the research was conducted in the absence of any commercial or financial relationships that could be construed as a potential conflict of interest.

## Footnotes

^1^The Kullback–Leibler (KL) divergence measures the difference between two distributions. If two distributions are the same, the KL-divergence becomes 0. In the LMDP, the control cost is defined by how certain control ***u*** affects on state transition probability.

^2^The L-1 norm of a vector ***x*** = (*x*_1_, …, *x*_*n*_)^T^ is the sum of the absolute value of the coordinate of ***x***, computed by ‖***x***‖_1_ = ∑_*i*_|*x*_*i*_|.

## Appendix

### Optimization of function approximation parameters

When the cost function is non-negative, the value function *v*(***x***) is also non-negative, and therefore, the inequality 0 ≤ *z*(***x***) ≤ 1 holds at any ***x*** by the definition of the desirability function (Equation 10). In order to satisfy this inequality, the constraint *w*_*i*_ ≥ 0 for all *i* is required since we assume that the basis function is a non-normalized Gaussian function. This constrained optimization on ***w*** is efficiently solved by the following quadratic programming

(29) $$\underset{w}{\min}\;e,\text{ s}.\text{t}.\;w_{i}\geq 0,\;\forall i.$$

To optimize **θ**, it is possible to apply the Levenberg–Marquardt algorithm to minimize the square error (Equation 18). However, it was reported that the desirability function become *z*(***x***_*n*_; ***w***, **θ**) ≈ 0 during the minimization process because the center position of the basis functions ***m***_*i*_ move away from collocation states ***x***_*n*_ (Todorov, 2009b). To avoid the trivial solution *z*(***x***) = 0, the following constraint is introduced,

(30) $$1^{\text{T}}F(\theta )w=\sum_{n=1}\hat{z}(x_{n};w,\theta )=\text{const}.$$

This constrained problem is optimized by the Levenberg–Marquardt algorithm. When we define  and ***g*** = ∂ (**1**^T^***F***(**θ**)***w***)/∂**θ**, then the objective function is given by  where **δ** and γ denote the gradient direction of the update rule and the parameter between 0 and 1, respectively. This is solved by the Lagrange multiplier methods.
